# Isosakuranetin-5-*O*-rutinoside: A New Flavanone with Antidepressant Activity Isolated from *Salvia elegans* Vahl.

**DOI:** 10.3390/molecules181113260

**Published:** 2013-10-25

**Authors:** Manáses González-Cortazar, Ana María Maldonado-Abarca, Enrique Jiménez-Ferrer, Silvia Marquina, Elsa Ventura-Zapata, Alejandro Zamilpa, Jaime Tortoriello, Maribel Herrera-Ruiz

**Affiliations:** 1 Southern Biomedical Research Center, Mexican Institute of Social Security, Argentina 1, Centro 62790, Xochitepec, Morelos, Mexico; E-Mails: gmanases@hotmail.com (M.G.-C.); ana_mazz@hotmail.com (A.M.M.-A.); enriqueferrer_mx@yahoo.com (E.J.-F.); azamilpa_2000@yahoo.com.mx (A.Z.); cibis_herj@yahoo.com.mx (M.H.-R.).; 2 Chemical Research Center, Universidad Autónoma del Edo. de Morelos (UAEM), Av. Universidad 1001, Col. Chamilpa 62210, Cuernavaca, Morelos, Mexico; E-Mail: smarquina21@hotmail.com; 3 Development Center Biotic Products, Instituto Politécnico Nacional (IPN), 62761 Yautepec, Morelos, Mexico; E-Mail: eventura@ipn.mx

**Keywords:** *Salvia elegans*, flavanone, rutinoside, isosakuranetin, NMR, ursolic acid, antidepressant activity

## Abstract

Ursolic acid (**1**) and a new flavanone, 5-*O*-(6-rhamnosylglucoside)-7-hydroxy-4'-methoxyflavanone (**2**), were isolated from the leaves of *Salvia elegans* Vahl. These natural products displayed antidepressant activity in mice as determined by means of a forced swimming test (FST) evaluation. Structural elucidation was carried out by chemical derivatization (acetylation) and spectroscopic analyses, such as ^1^H- and ^13^C-NMR and two-dimensional (2-D) COSY, heteronuclear multiple quantum coherence (HMQC), and heteronuclear multiple-bond correlation (HMBC) spectroscopy experiments.

## 1. Introduction

Depression is a psychiatric disorder characterized by a decrease in the ability of individuals to experience pleasure and by a depressed mood; it is a chronic and disabling mental illness that causes high morbidity and mortality [1]. The World Health Organization (WHO) estimate that there are about 350 million people with depression and predicts that by 2020, this disorder will be the second leading cause of disability worldwide, calculated for all ages and both genders [2]. Today, depression is already the second leading cause of days lost due to disability (DALYs) in the 15–44 years-of-age group for both genders. Despite the existence of treatments such as antidepressants that selectively inhibit serotonin reuptake, tricyclic antidepressants, and monoamine oxidase inhibitors, (MAOIs), among others, all of these have major side effects. Furthermore, improvement is needed in the treatment of patients with depression, because therapeutic effectiveness is only observed in about 45% of patient after a prolonged period of drug consumption, which leads to treatment discontinuation; in addition, 15% of these patients do not respond to current therapies [3]. Depression diminishes the Quality of life (QOL) of patients because it deteriorates their physical and mental health. The results of a study conducted in 2007 through a worldwide health survey, in which 245,404 cases from different regions around the world were analyzed, showed alarming data: pure depressive disorders were found in 3.2% of cases that, within a range of 9.3%–23.0% of participants with one or more chronic physical diseases such as arthritis and diabetes, among others, have a high rate of comorbidity with depression. Depression was found to have the largest value in an index of health worsening when compared to other chronic conditions; it was also observed that the comorbidity of this psychiatric disorder with chronic diseases causes a worse state of health than when chronic diseases occur alone [4]. This fact has led to an intensification of the search for new pharmacological treatments, and medicinal plants have become a source of study for the development of new therapeutic strategies.

The Lamiaceae family comprises about 220 genera and 4,000 species. One of the largest genera of the family is *Salvia* L., represented by >900 species that are widely distributed in various regions of the world [5,6]. In Mexico, different species of *Salvia* are used in Mexican Traditional Medicine to treat >60 conditions, ranging from pain to central nervous system (CNS) disorders; they are employed as a tranquilizer, a hypnotic, a sedative, and as an antiepileptic [7,8,9,10]. The main compounds found in the leaves of this family are flavonoids and terpenoids [10,11,12,13].

In particular, the aerial parts of the species *Salvia elegans* Vahl. are widely used by Mexicans to treat CNS disorders, mainly anxiety. This plant is a shrub that can measure between 80 cm and 2 m in height and that has a hairy stem with wet hairs on the young plant parts. It has red pipe-shaped flowers that open into two lips. It is native to Mexico and grows in temperate climates between 2,280 and 3,100 meters above sea level (MASL) in oak, pine, and mixed forests [9].

Of the reported studies, there is one indicating that the methanolic extract of this species is able to displace [3H]-(N)-scopolamine from the muscarinic receptors located in the membranes of human cerebral cortex cells [14]. In agreement with its ethnomedical use, it was reported that the hydroalcoholic extract (60%) of *S. elegans* leaves and flowers exerts an anxiolytic and antidepressant effect in both mice [15] and rats [16], utilizing the plus-maze and forced swimming test (FST) models, respectively. Moreover, this extract and its fraction, which contains flavonoids and phenylpropanoids, induce an antihypertensive effect by inhibiting the angiotensin-converting enzyme (ACE) and by acting as an angiotensin II (AG II) antagonist [17].

After chemical analysis of *S. elegans*, it has been possible to isolate the following triterpenic acids: ursolic, oleanolic, and 3β,23-dihydroxy-12-en-28-oic, as well as a flavonoid identified as 3-acetoxy-7-methoxyflavone [18].

The pharmacological and chemical study of the species has been ongoing, based on the previously published reports. In this work, the main objective was to perform chemical fractionation by using chromatography and spectroscopy methods, guided by the antidepressant activity evaluated with the FST in ICR male mice, to achieve the isolation and structural elucidation of the compound responsible for the antidepressant activity.

## 2. Results and Discussion

### 2.1. Evaluation of the Hydroalcoholic Extract

**Figure 1:**
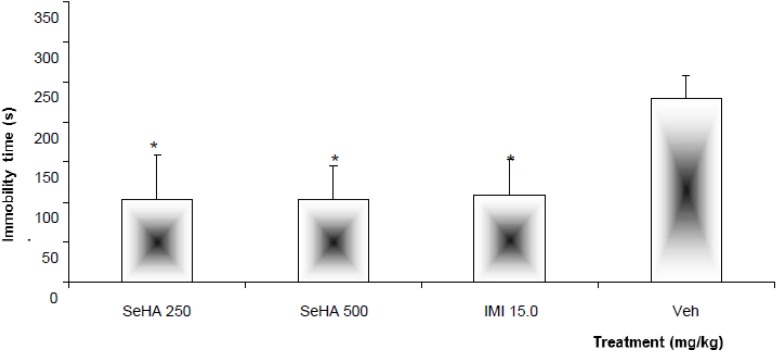
Effect produced by oral administration of the *Salvia elegans* hydroalcoholic extract (SeHA, 250 and 500 mg/kg) and comparison with the treatment with Imipramine (IMI, 15 mg/kg) and Vehicle (Veh, 1.0% Tween [TW] 20) on immobility time in FST. Statistical analysis was carried out with Analysis of variance (ANOVA) followed by the *post hoc* Dunnett test (*n* = 8: mean ± standard deviation [SD]) (* *p* < 0.05).

As illustrated in Figure 1, oral administration of the hydroalcoholic extract obtained from *S. elegans* (SeHA) at doses of 250 and 500 mg/kg was able to reduce the immobility time in the FST. This effect was not different (*p* > 0.05) from that produced by the treatment with imipramine (IMI, 15 mg/kg), considered as the positive control group. However, both treatments were statistically (*p* < 0.05) different with respect to the negative control group, which was administered the vehicle (Veh, 1.0% Tween 20 solution). The effect produced by both doses (250 and 500 mg/kg) of SeHA was similar, probably because they were in the same range of effectiveness.

### 2.2. Chromatographic Separation of the Hydroalcoholic Extract and Isolation and Elucidation of Ursolic Acid *(**1**)* and a Flavanone ***2***

**Figure 2:**
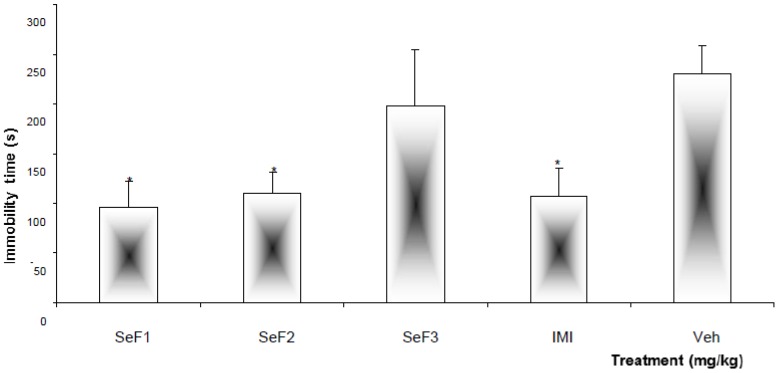
Effect produced by different sub-fractions, SeF1, SeF2, and SeF3 (orally, 250 mg/kg) obtained from *Salvia elegans*, as well as imipramine (IMI, 15 mg/kg, intraperitoneally [i.p.]) and the vehicle (Veh, 1.0% Tween [TW] 20) administered to mice in the FST. Statistical analysis was carried out with Analysis of variance (ANOVA) followed by the *post hoc* Dunnett test (*n* = 8; mean ± standard deviation [SD]) (* *p* < 0.05).

Fractions obtained from the chemical separation of SeHA were evaluated at a single dose of 250 mg/kg using the FST. The least polar fraction (SeF3) did not modify the behavior of animals in comparison with the mice administered the vehicle, while the more polar fractions (SeF1 and SeF2) were able to induce a significant (*p* < 0.05) reduction of immobility time in FST (Figure 2). The effect produced by both fractions was similar to that exhibited by IMI.

The SeF1 active fraction was then separated into three sub-fractions. The first two lesser polar sub-fractions (F1-1 and F1-2) at a dose of 50 mg/kg did not produce changes in the behavior of mice subjected to FST when compared with those of the negative control group treated with the Veh (Table 1). At the same time, F1-3 (single dose of 50 mg/kg) was able to induce a significant (*p* < 0.05) reduction of immobility in FST (Table 1). Then, this sub-fraction was resubmitted to chemical separation, and a considerable amount of ursolic acid (**1**) was isolated and purified. This product was evaluated in FST at doses of 15 and 30 mg/kg without noting a decrease of immobility time. Structural identification of this acid was directly compared with a previously isolated sample [18]. Ursolic acid does not seem to be responsible for the effect produced by F1-3 on FST. This activity may be due to another non- identified minor compound present in this fraction.

**Table 1:** Effect produced by different fractions and compounds separated from SeF1 and SeF2 on immobility time in FST.

Treatment	Dose (mg/kg)	Immobility time
SeF1	250	96.2 ± 26.1 *
F1-1	50.0	273.1 ± 12.1
F1-2		261.7 ± 19.6
F1-3		113.8 ± 23.6 *
UA	15.0	235.5 ± 1.9
	30.0	221.2 ± 29.6
SeF2	250.0	110.3 ± 21.0 *
F2-1	50.0	286.5 ± 9.2
F2-2		241.8 ± 7.5
Isosakuranetin-5- *O*-rutinoside	15.0	122.0 ± 19.6 *
IMI	15.0	107.9 ± 23.4 *
Veh (TW 20, 1%)	100 µL/10 g	230.2 ± 28.2

Data presented as means ± standard deviation (SD) (*n* = 8). * p < 0.05 compared with the negative control group by using Analysis of Variance (ANOVA) followed by the *post hoc* Dunnett test. FST = Forced swimming test; IMI = Imipramine; UA = Ursolic acid; Veh = Vehicle; TW = Tween.

**Figure 3:**
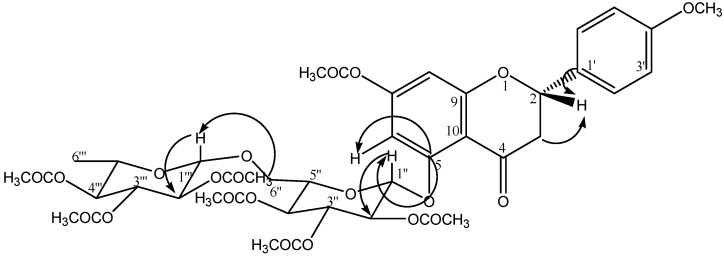
HMBC correlations for C-3, C-5, C-1', H-2, H-1″ and H-1‴.

**Figure 4:**
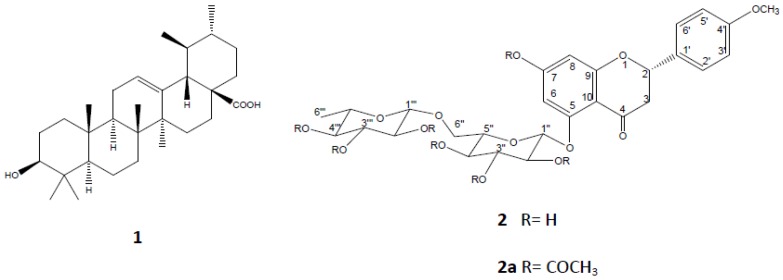
Ursolic acid (**1**), isosakuranetin-5-*O*-rutinoside (**2**), and isosakuranetin-5*O*-rutinoside heptaacetate (**2a**).

On the other hand, the sub-fractions obtained from SeF2: F2-1 and F2-2 were also tested in the FST, without exhibiting differences with respect to the negative control group. F2-1 was not subjected to the fractionation process because the low yield. However, F2-2 was fractionated and a flavonoid (an amorphous solid) was isolated (compound **2**). In the positive-ion fast atom bombardment mass spectrum (FAB-MS) of **2**, quasimolecular ion peaks at *m/z* 594 [M]^+^ were observed, and high-resolution fast atom bombardment mass spectroscopy (HRFABMS) analysis revealed that the molecular formula corresponded to C_28_H_34_O_14_ (*m/z* = 594.57 [M]^+^). The aglycone was identified as isosakuranetin [5,7-dihydroxy-4'-methoxyflavanone] based on distortionless enhancement polarization transfer (DEPT), correlation spectroscopy (COSY), heteronuclear multiple quantum coherence (HSQC), and heteronuclear multiple-bond correlation (HMBC) spectra of its peracetate derivative **2a**. The ^1^H-, ^13^C-NMR spectra of **2a** showed three systems: an aromatic AB system [δ 6.47 (1H, d, *J* = 2.4 Hz) and 6.30 (1H, d, *J* = 2.4 Hz)]; another aromatic A_2_B_2_ system [δ 7.37 (2H, d, *J* = 8.8 Hz) and 6.94 (2H, d, *J* = 8.8 Hz)], and an ABX system for the H-2 and H-3 signals [δ 5.45 (1H, dd, *J* = 2.8, 13.2 Hz), 3.04 (1H, dd, *J* = 13.2, 16.4 Hz), 2.74 (1H, dd, *J* = 2.8, 16.8 Hz), respectively, indicating an isosakuranetin flavanone nucleus; the remaining signals correspond to two sugars. Two anomeric protons [δ 5.19 (1H, d, *J* = 6.8 Hz, glucosyl H-1″) and δ 4.69 (1H, d, *J* = 0.8 Hz, rhamnosyl H-1″′) ] together with signals at δ 3.62 (1H, dd, *J* = 4.8, 11.6 Hz, glucosyl H-6a″), 3.79 (1H, dd, *J* = 2.8, 11.6 Hz, glucosyl H-6b″) and 1.14 (3H, d, *J* = 6.4 Hz, rhamnosyl H-6″′) revealed a rutinoside moiety. The position of the sugar residue in **2a** was defined unambiguously to be at C-5 due to the long-range correlation between C-5 (δ 162) of the isosakuranetin and H-1″ (δ 5.19, d*, J* = 6.8 Hz) of the glucopyranosyl unit (Figure 3). Based on this information, the natural product was identified as isosakuranetin-5-*O*-rutinoside heptacetate (**2a**), a novel flavanone in *S. elegans* (Figure 4). Direct comparison of spectroscopic data from this compound displayed high similarity with those previously described for isosakuranetin-7-*O*-rutinoside [19,20,21], except for the sugar, which is linked to the 5-hydroxy position of isosakuranetin.

Isosakuranetin-5-*O*-rutinoside (**2**) was tested at a dose of 15 mg/kg. This compound demonstrates its ability to significantly reduce immobility time in the FST (*p* < 0.05) (Table 1). In this work, it is presented, to our knowledge for the first time, as an isolated compound from *S. elegans* that exhibited antidepressant activity; there are no previous chemical or pharmacological data regarding this metabolite. Even more so, there is no pharmacological evidence concerning any activity of the genin (isosakuranetin) on the CNS.

## 3. Experimental

### 3.1. General Experimental Procedures

Melting points were obtained by means of a Thermo Scientific IA9000 series melting point apparatus and were uncorrected. All NMR spectra were recorded on Varian INOVA-400 at 400 MHz for ^1^H-NMR, Nuclear Overhauser enhancement spectroscopy (NOESY), ^1^H-^1^H for COSY, HSQC, and HMBC, and 100 MHz for ^13^C-NMR and DEPT in CDCl_3_. Chemical shifts are reported in parts per million (ppm) relative to tetramethylsilane (TMS). Mass spectrometry was performed by means of Hewlett Packard 5985-B and JEOL-AX 505 HA instruments. High-performance liquid chromatography (HPLC) analysis was implemented with a Waters HPLC instrument equipped with a Waters 996 UV photodiode array detector (900) set at 210 nm, using a Lichrosphere® 12.5 × 4 × 5 mm column at a flow rate of 1.0 mL/min, and an isocratic system of CH_3_CN-H_2_O (70:30) as the mobile phase. For quantification of ursolic acid (UA), a standard product (Sigma-Aldrich, St. Louis, MO, USA) was dissolved at a proportion of 5 mg/mL in methanol, and different dilution series (25, 50, 100, and 200 mg/mL) were used.

### 3.2. Plant Material

*Salvia elegans* Vahl. (Lamiaceae) leaves were collected from Ozumba, state of Mexico, which is North of Mexico City (2,340 m.a.s.l.), in November 2010. Some samples were prepared for identification by Margarita Avilés and Macrina Fuentes, Researchers from Botanical Garden at National Institute of Anthropology and History, Cuernavaca, Morelos, Mexico, where some vouchers were deposited for future reference (INAHM-2034).

### 3.3. Extraction and Isolation of Ursolic acid *(**1**)* and Isosakuranetin-5-O-Rutinoside *(**2**)*

Leaves were selected from the collected plants and were dried under dark conditions at room temperature; this material (2.5 kg) was successively extracted with 60% ethanol for 2 h at 50 °C for three times (each time with 13 L of the solvent). The hydroalcoholic extract obtained was evaporated to dryness with a rotary evaporator under reduced pressure, producing a 420 g residue. The hydroalcoholic extract was resuspended in 1.5 L of distilled water and successively extracted with *n*-hexane (2 L, SeF1) and butanol (2 L, SeF2). The fractions were evaporated to dryness, obtaining a yield of 5.4 g for SeF1 and one of 25 g for SeF2. The remaining aqueous layer was lyophilized in order to obtain the water soluble fraction (63.5 g), which was identified as SeF3. All fractions were submitted to evaluation by means of FST.

The SeF1 fraction (3 g) was adsorbed in silica gel and applied to a silica gel column (90 g, 70–230 mesh; Merck, Darmstadt, Germany). A gradient of *n*-hexane-ethyl acetate-methanol was utilized to elute the column; 24 fractions of 125 mL each were collected. The obtained portions were reunited according to their similarity in Thin-layer chromatography (TLC) in three groups (F1-1, F1-2, and F1-3). It is noteworthy that F1-3 on TLC showed a single spot, which corresponded to UA (**1**, 300 mg). To corroborate the identity of this compound, it was compared with a standard sample.

SeF2 (64.8 g, 15.42%) was separated employing a chromatographic column with silica gel (350 g, 70–230 mesh; Merck) and eluted with a solvent mixture with increasing polarity (dichloromethane-methanol); 84 fractions of 250 mL each were collected. Fractions 46–49 (SeF2-2) were combined and recrystallized; then, a solid amorphous compound (37.2 mg) was obtained and denominated Isosakuranetin-5-*O*-rutinoside (**2**), which was elucidated by the following chemical derivatization (acetylation):

*5-O-(6-Rhamnosylglucoside)-7-hydroxy-4'-methoxyflavanone, Isosakuranetin-5-O-rutinoside* (**2**); mp 210–212 °C; λ_nm_ 226.3, 282.8, 329.1; HRFABMS, *m/z* 594[M]^+^; calcd for C_28_H_34_O_14_, 594.57.

### 3.4. Acetylation of Isosakuranetin-5-O-rutinoside *(**2**)*

Compound **2** (30 mg) was treated with Ac_2_O (3 mL) and pyridine (1 mL) for 2 h [22]. The reaction was stopped with ice (5 g), afterward, a precipitate was obtained. This product was filtered and washed with water to render a white solid compound **2a** (28.3 mg). The structure of this compound was identified based on one-dimensional (1-D) and 2-D NMR techniques and by comparison with certain data in the literature [19,20,21].

*Isosakuranetin-5-O-rutinoside heptaacetate* (**2a**) (28.3 mg, 75.4%); mp 130–132 °C; ^1^H-NMR (400 MHz, CDCl_3_ ); δ 5.45 (1H, dd, *J*= 2.8, 13.2 Hz, H-2), 3.04 (1H, dd, *J* = 13.2, 16.4 Hz, H-3a), 2.74 (1H, dd, *J* = 2.8, 16.8 Hz, H-3b), 6.47 (1H, d, *J* = 2.4 Hz, H-6), 6.30 (1H, d, *J* = 2.4 Hz, H-8), 7.37 (2H, d*, J* = 8.8 Hz, H-2', H-6'), 6.94 (2H, d, *J* = 8.8 Hz, H-3', H-5'), 3.82 (3H, s, OCH_3_), 2.38 (3H, s, OCOCH_3_), 2.09 (3H, s, OCOCH_3_), 2.08 (3H, s, OCOCH_3_), 2.07 (3H, s, OCOCH_3_), 2.03 (6H, s, OCOCH_3_), 1.95 (3H, s, OCOCH_3_), 5.19 (1H, d, *J* = 6.8 Hz, H-1″), 5.03-5.13 (3H, m, H-2″, H-3″, H-4″), 5.02 (1H, dd, *J* = 9.6, 9.6 Hz, H-5″), 3.62 (1H, dd, *J* = 4.8, 11.6 Hz, H-6a″), 3.79 (1H, dd, *J* = 2.8, 11.6 Hz, H-6b″), 4.69 (1H, d, *J* = 0.8 Hz, H-1‴), 5.26–5.21 (3H, m, H-2‴, H-3‴, H-4‴), 3.85-3.88 (1H, m, H-5‴), 1.14 (3H, d, *J* = 6.4 Hz, H-6‴); ^13^C-NMR (100 MHz, CDCl_3_); δ 79.4 (CH, C-2), 44.7 (CH_2_, C-3), 189.3 (C, C-4), 162 (C, C-5), 102.3 (CH, C-6), 152 (C, C-7), 105.9 (CH, C-8), 164.1 (C, C-9), 109.7 (C, C-10), 130.3 (C, C-1'), 128 (2CH, C-2', C-6'), 114.3 (2CH, C-3', C-5'), 160.2 (C, C-4'), 55.5 (CH_3_, OCH_3_), 169.3 (C, OCOCH_3_), 169.5 (C, OCOCH_3_), 169.7 (C, OCOCH_3_), 170 (2C, OCOCH_3_), 170.2 (C, OCOCH_3_), 170.4 (C, OCOCH_3_), 21.2 (7CH_3_, OCOCH_3_), 97.7 (CH, C-1″), 71 (CH, C-2″), 69.5 (CH, C-3″), 68.7 (CH, C-4″), 73.3 (CH, C-5″), 66 (CH_2_, C-6″), 98.1 (CH, C-1‴), 72.5 (CH, C-2‴), 69.1 (CH, C-3‴), 70.9 (CH, C-4‴), 66.8 (CH, C-5‴), 17.4 (CH_3_, C-6‴).

### 3.5. Drugs

Imipramine (IMI, 15 mg/kg; Sigma-Aldrich), Ursolic acid (UA, Standard reference, Sigma-Aldrich,), Ursolic acid (UA, isolated from *S. elegans*), and Isosakuranetin-5-*O*-rutinoside (**2**, isolated from *S. elegans*), Tween 20 solution (TW 20, Merck, Darmstadt, Germany).

### 3.6. Animals and Drug Administration

Male ICR mice with a weight around 35 g were used. All animals were purchased from Harlan (Mexico, City) and maintained for 3 weeks in an animal house with a 12h:12h light-darkness cycle and free access to water and food (pellets, Harlan). Three days before testing began, the animals were conditioned to the laboratory environment and to the researcher. All experiments were carried out between 8:00 and 13:00 h and conducted in accordance with the Federal Regulations for Animal Experimentation and Care (Ministry of Agriculture, NOM-062-ZOO-1999, Mexico, City). The experimental protocols were approved by the Research Committee of Mexican Institute of Social Security (FIS/IMSS/PROT/558). The minimal number of animals and minimal duration of observation required to obtain consistent data were employed. Groups of eight mice per treatment were used and each group was administered by oral pathway (24, 18, and 1 h prior to the experimental session for the FST for at a constant volume of 300 mL).

With the exception of IMI, which was administered i.p., all of the following treatments were administered orally: fraction SeHA at doses of 250 and 500 mg/kg; fractions SeF1, SeF2, and SeF3 at a single dose of 250 mg/kg, and the sub-fractions obtained from active fractions F1-1, F1-2, F1-3, F2-1, and F2-2, at a single dose of 50 mg/kg, with the isolated compound (**1**) at doses of 15 and 30 mg/kg and compound (**2) **at a dose of 15 mg/kg. IMI was used as positive control drug (IMI, 15 mg/kg).

### 3.7. Forced Swimming Test (FST)

The forced swimming test (FST) is a widely used pharmacological *in vivo* model for assessing antidepressant activity. The development of immobility, when mice are placed in an inescapable cylinder filled with water, reflects the cessation of persistent escape-directed behavior. The apparatus consisted of a clear Plexiglas cylinder (20 cm in height × 12 cm in diameter) filled to a 15-cm depth with water (24 ± 1 °C). In the pre-test session, each animal was placed individually into the cylinder for 15 min, 24 h prior to the 5-min swimming test. During the test session, a trained observer registered immobility time, considered when the mouse made no further attempts to escape, aside from the movements necessary to maintain its head above water. Immobility reflected a state of lowered mood in which the animals had given up hope of finding an exit and had resigned themselves to the experimental situation [23].

### 3.8. Data Analysis

Results from antidepressant activity were statistically analyzed with Analysis of variance (ANOVA) followed by the *post hoc* Dunnett test with a significance level of *p* ≤ 0.05. SPSS ver. 11.0 software program was employed for this purpose.

## 4. Conclusions

In summary, *Salvia elegans* produces ursolic acid (UA, **1**), which was identified in fraction F1-3. This compound was isolated from the aerial parts of the plant using chromatographic techniques. Additionally, from the F2-2 fraction, a new flavanone was isolated, which structural elucidation identified as isosakuranetin-5-*O*-rutinoside (**2**). In this work we have presented, to our knowledge for as the first time, this compound, which demonstrated to possess antidepressant activity. The correct structure was assigned by NMR detailed spectroscopy analysis.

## Supplementary Materials

Supplementary materials can be accessed at: http://www.mdpi.com/1420-3049/18/11/13260/s1.

## References

[1] Gaffrey MS., Luby JL., Barch DM. (2013). Towards the study of functional brain development in depression: an interactive specialization approach. Neurobiol. Disease.

[2] World Health Organization (WHO). http://www.who.int/mediacentre/news/notes/2012/mental_health_day_20121009/en/.

[3] Nash J., Nutt D. (2007). Antidepressants. Psychiatry.

[4] Moussavi S., Chatterji S., Verdes E., Tandon A., Patel V., Ustun B. (2007). Depression, chronic diseases, and decrements in health: Results from the World Health Surveys. Lancet.

[5] Rosua J.L., Blanca G. (1986). Revisión del género *Salvia* L. (*Lamiaceae*) en el mediterráneo occidental: La sección de *Salvia*. Acta Bot. Malacit..

[6] Rodríguez-Hahn L., Cárdenas J. (1999). Comparative chemotaxonomy in Labiatae. Curr. Top. Phytochem..

[7] Foster S., Tyler VE. (2000). Tyler’s Honest Herbal: A sensible guide to the use of herbs and related remedies.

[8] Steinegger E., Hänsel R. (1988). Lehrbuch der Pharmakognosie und Phytopharmazie.

[9] Aguilar A., Camacho J.R., Chino S., Vázquez P., López P. (1994). Herbario Medicinal del Instituto Mexicano del Seguro Social.

[10] Topҫu G. (2006). Bioactive triterpenoids from *Salvia* species. J. Nat. Prod..

[11] Pereda-Miranda R., Delgado G. (1986). Flavonoids from *Salvia nicolsoniana*. J. Nat. Prod..

[12] Esquivel B., Vergara F., Matus W., Hernández-Ortega S., Ramírez-Apán MT. (2005). Abietane diterpenoids from the roots of some Mexican *Salvia* species (Labiatae). Chemical diversity, phytogeographical significance and cytotoxic activity. Chem. Biodivers..

[13] Jaime-Vasconcelos M.A., Frontana-Uribe B.A., Morales-Serna J.A., Salmón M., Cárdenas J. (2011). Structure of salvioccidentalin, a diterpenoid with a rearranged neo-clerodane skeleton from *Salvia occidentalis*. Molecules.

[14] Wake G., Court J., Pickering A., Lewis R., Wilkins R., Perry E. (2000). CNS acetylcholine receptor activity in European medicinal plants traditionally used to improve failing memory. J. Ethnopharmacol..

[15] Herrera-Ruiz M., García-Beltrán Y., Mora S., Díaz-Véliz G., Viana GS., Tortoriello J., Ramírez G. (2006). Antidepressant and anxiolytic effects of hydroalcoholic extract from *Salvia elegans*. J. Ethnopharmacol..

[16] Mora S., Millán R., Lungenstrass H., Díaz-Véliz G., Morán JA., Herrera-Ruiz M., Tortoriello J. (2006). The hydroalcoholic extract of *Salvia elegans* induces anxiolytic- and antidepressant-like effects in rats. J. Ethnopharmacol..

[17] Jiménez-Ferrer E., Hernández F.H., González-Cortazar M., Tortoriello J., Herrera-Ruiz M. (2010). Antihypertensive activity of *Salvia elegans* Vahl. (Lamiaceae): ACE inhibition and angiotensin II antagonism. J. Ethnopharmacol..

[18] Marquina S., García Y., Álvarez L., Tortoriello J. (2008). 3-Acetoxy-7-methoxyflavone, a novel flavonoid from anxiolytic extract of *Salvia elegans* (Lamiaceae). Nat. Prod. Commun..

[19] Yueh-Hsiung K., Shu-Mei L., Jeng-Shiow L. (2000). Constituents of the whole herb of *Clinoponium laxiflorum*. J. Chin. Chem. Soc..

[20] Wagner H., Hörhammer L., Aurnhammer G., Farkas L. (1968). Strukturaufklärung und synthese des didymins, eines isosakuranetin-7-β-rutinosids aus *Monarda didyma* L.. Chem. Ber..

[21] Doganca S., Ulubelen A., Tuzlaci E. (1989). Flavanones from *Cyclotrichium niveum*. Phytochemistry.

[22] González M., Zamilpa A., Marquina S., Navarro V., Álvarez L. (2004). Antimycotic spriostanol saponins from *Solanum hispidum* leaves and their structure-activity relationships. J. Nat. Prod..

[23] Porsolt R.D., Bertin A., Jalfre M. (1977). Behavioral despair in mice: A primary screening test for antidepressants. Arch. Int. Pharmacodyn. Ther..

