# Supplementary material for: Isosakuranetin-5-O-rutinoside: A New Flavanone with Antidepressant Activity Isolated from Salvia elegans Vahl
Source: Molecules. 2013 Oct 25;18(11):13260–70. doi: 10.3390/molecules181113260 (PMC6270368; doi:10.3390/molecules181113260)

# Supplementary Materials

**Figure S1.** $^1$H-NMR ($CD_3OD$, 400MHz) compound **2**.

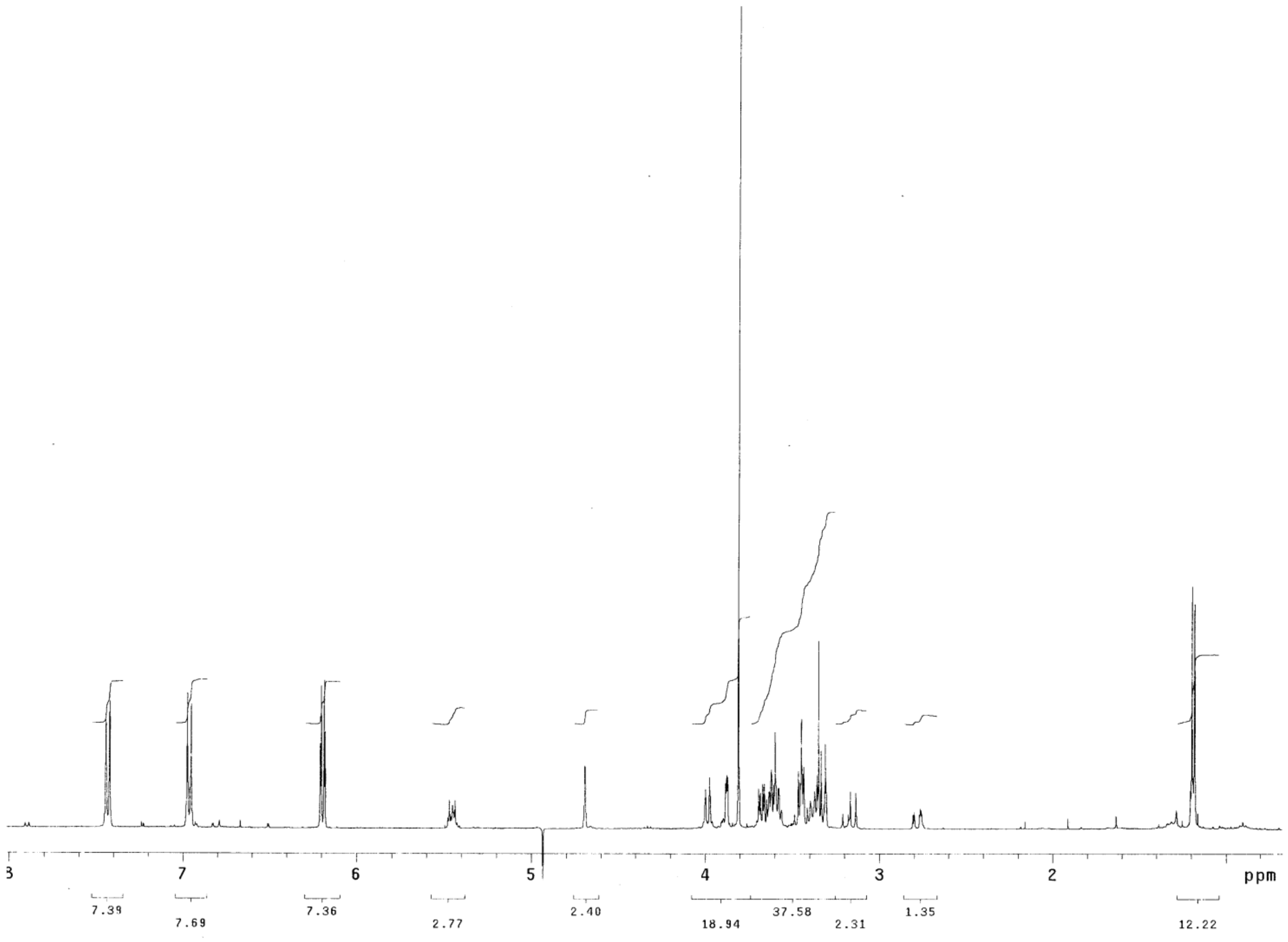

**Figure S2.** $^1$H-NMR ($CDCl_3$, 400MHz) compound **2a**.

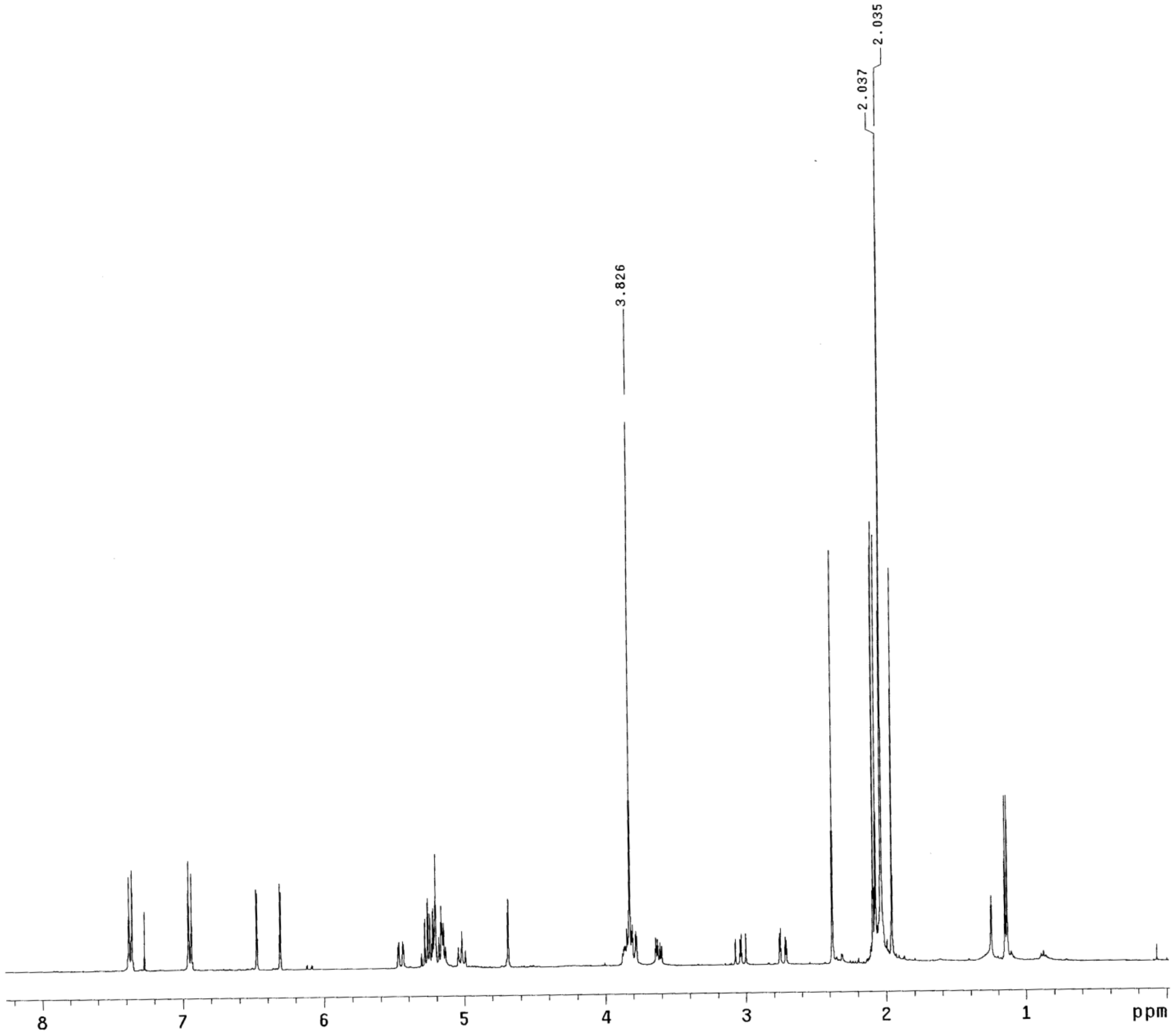

**Figure S3.** $^{13}$C-NMR ($CDCl_3$, 100MHz) compound **2a**.

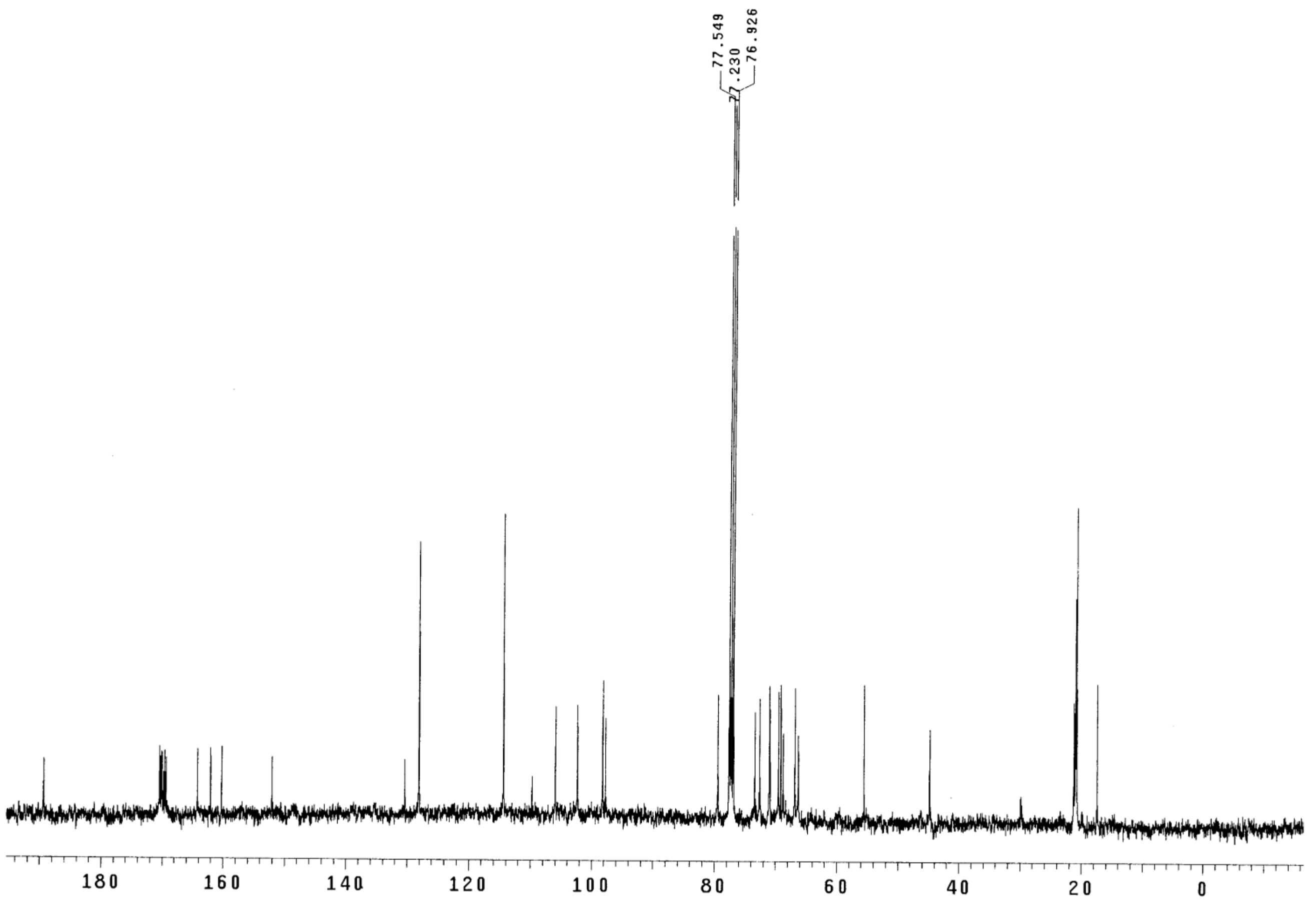

**Figure S4.** $^1H$-$^1H$ COSY ($CDCl_3$, 400MHz) compound **2a**.

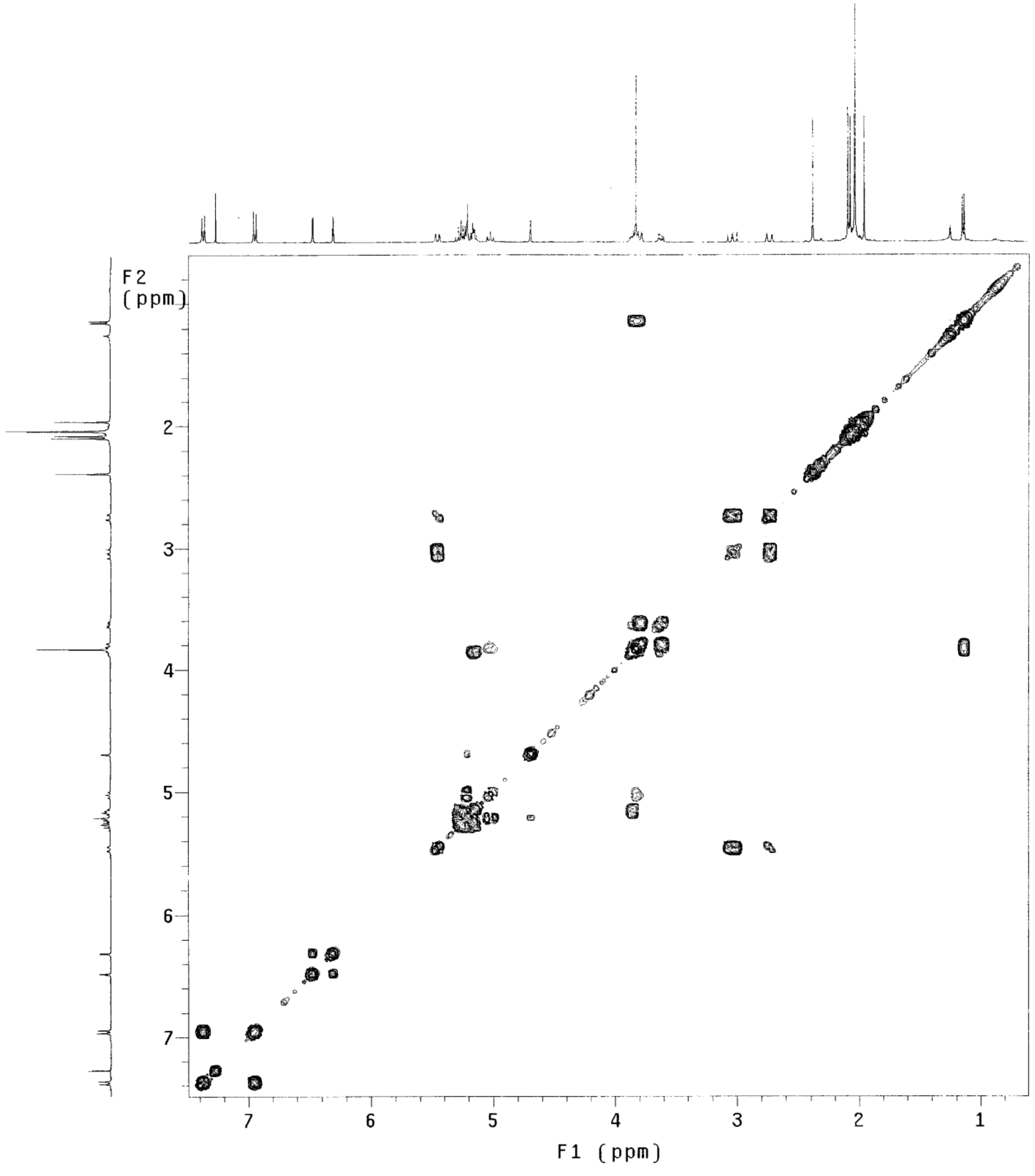

**Figure S5.** HSQC ($CDCl_3$, 400MHz) compound **2a**.

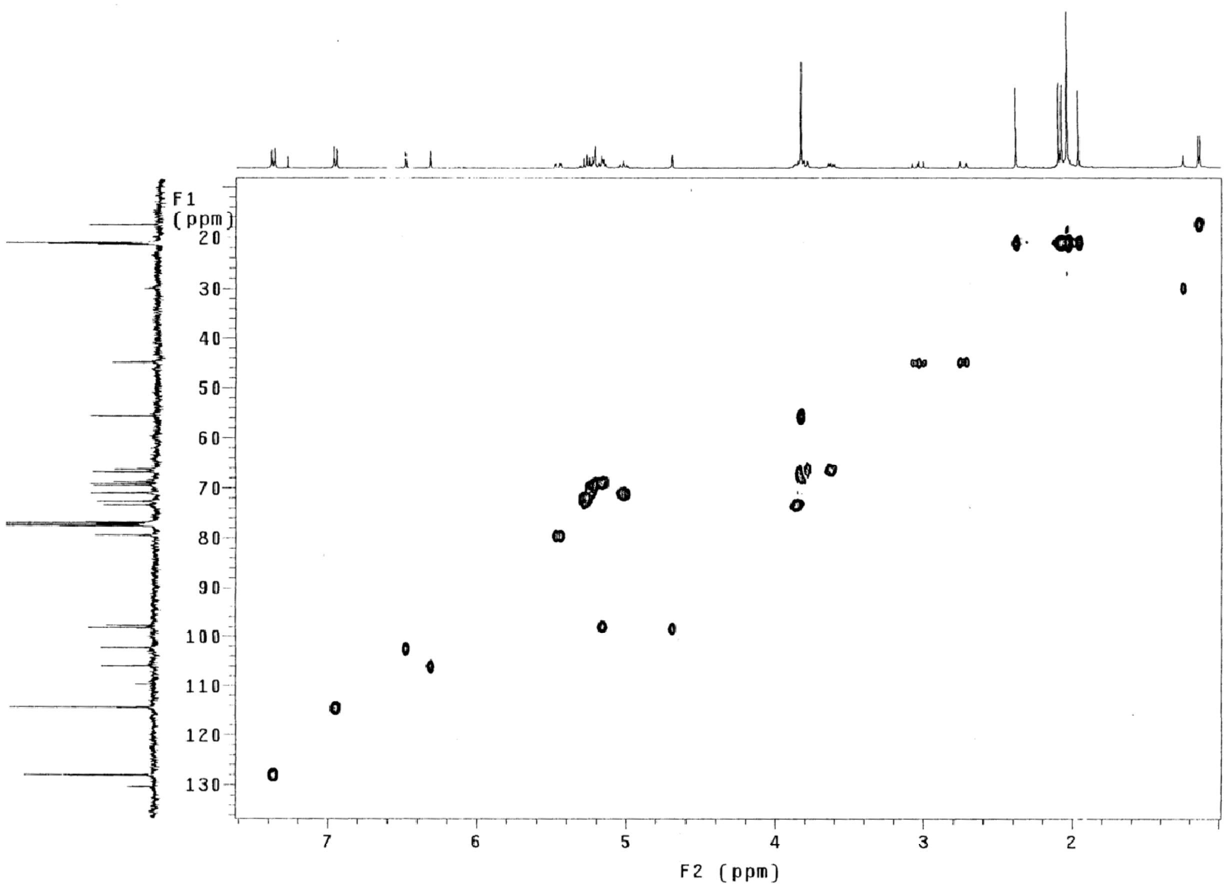

**Figure S6.** HMBC ($CDCl_3$, 400MHz) compound **2a**.

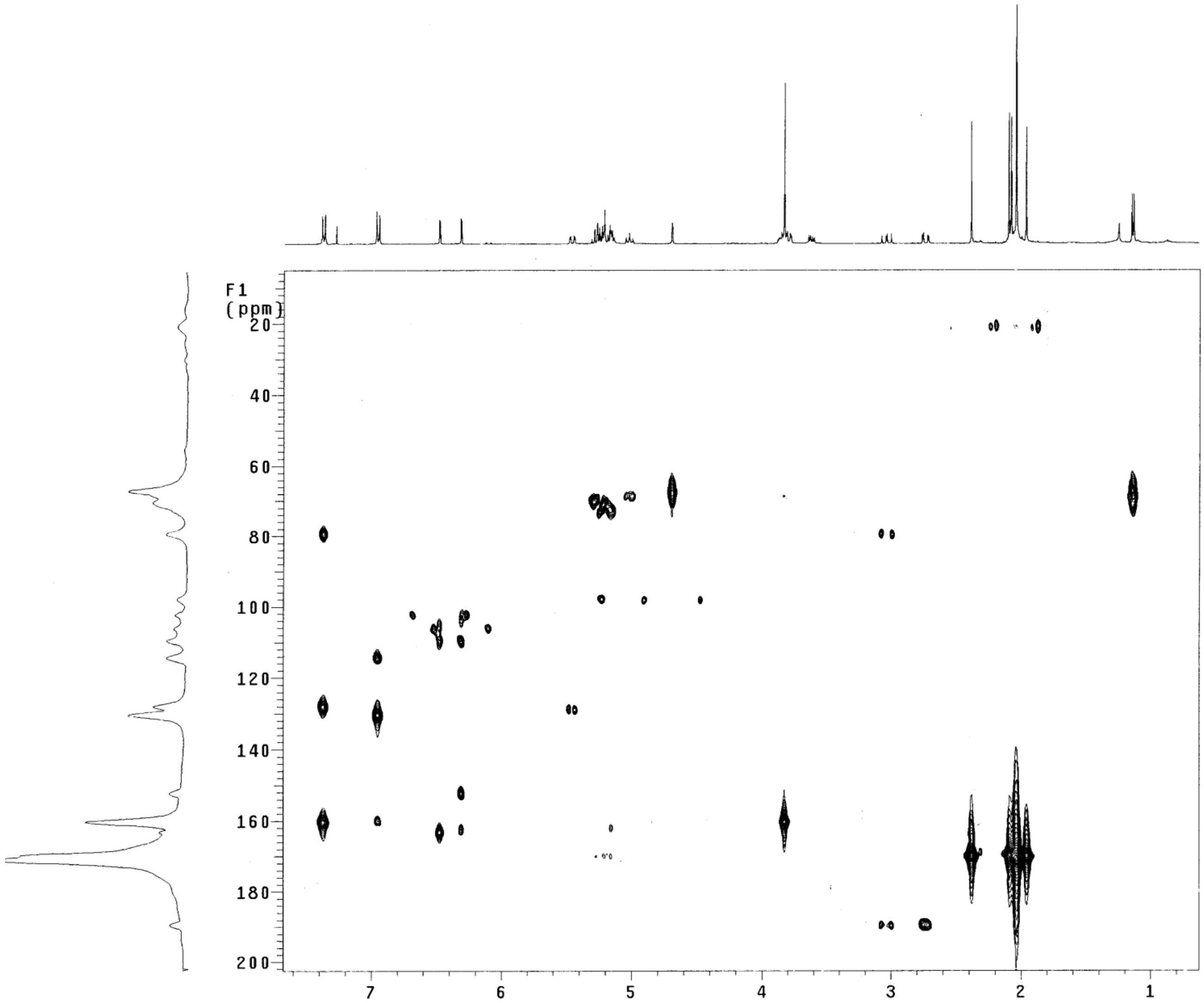

**Figure S7.** Mass spectra compound **2a**.

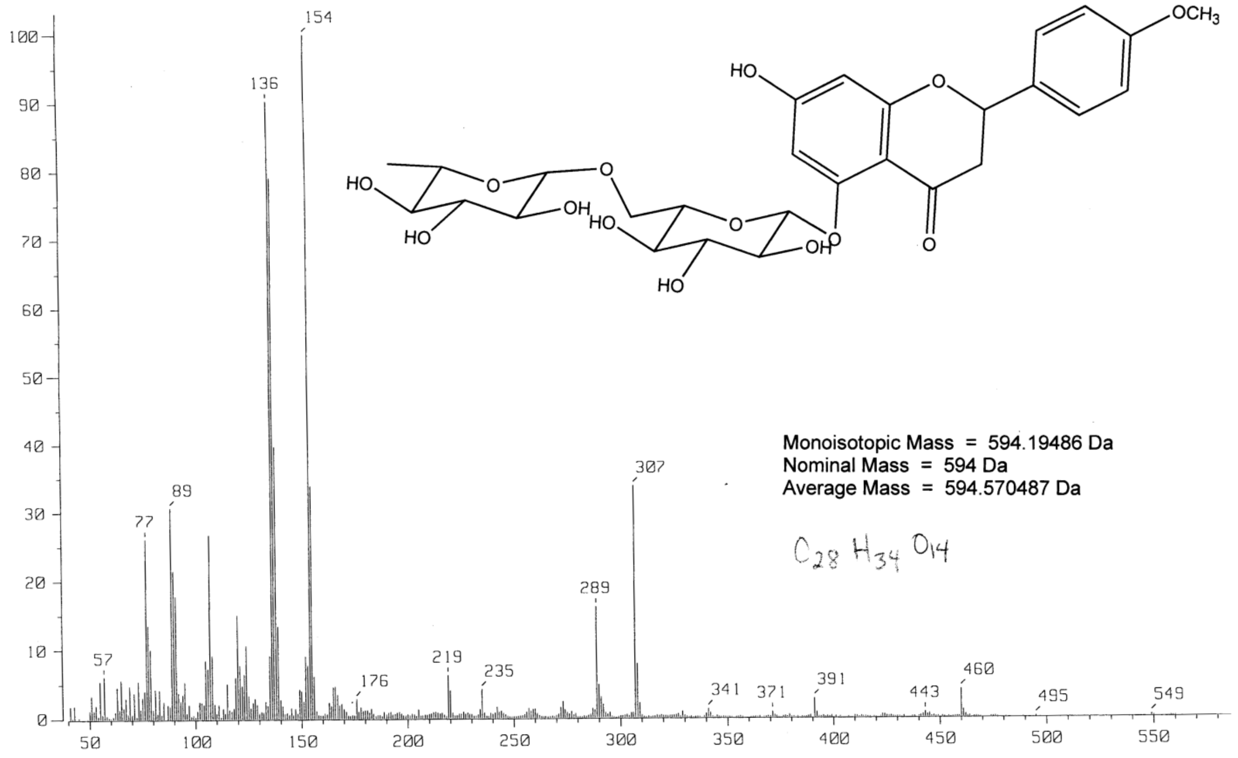

Supplement: Supplementary file 1 [file molecules-18-13260-s001.pdf]
